# Texture analysis of apparent diffusion coefficient maps: can it identify nonresponse to neoadjuvant chemotherapy for additional radiation therapy in rectal cancer patients?

**DOI:** 10.1093/gastro/goae035

**Published:** 2024-04-22

**Authors:** Qianyu Wu, Yongju Yi, Bingjia Lai, Jiao Li, Yanbang Lian, Junhong Chen, Yue Wu, Xinhua Wang, Wuteng Cao

**Affiliations:** Department of Radiology, The Sixth Affiliated Hospital, Sun Yat-sen University, Guangzhou, Guangdong, P. R. China; Guangdong Provincial Key Laboratory of Colorectal and Pelvic Floor Diseases, Guangdong Research Institute of Gastroenterology, The Sixth Affiliated Hospital, Sun Yat-sen University, Guangzhou, Guangdong, P. R. China; Biomedical Innovation Center, The Sixth Affiliated Hospital, Sun Yat-sen University, Guangzhou, Guangdong, P. R. China; Guangdong Provincial Key Laboratory of Colorectal and Pelvic Floor Diseases, Guangdong Research Institute of Gastroenterology, The Sixth Affiliated Hospital, Sun Yat-sen University, Guangzhou, Guangdong, P. R. China; Biomedical Innovation Center, The Sixth Affiliated Hospital, Sun Yat-sen University, Guangzhou, Guangdong, P. R. China; Department of Information Center, The Sixth Affiliated Hospital, Sun Yat-sen University, Guangzhou, Guangdong, P. R. China; Department of Radiology, Sun Yat-sen Memorial Hospital, Sun Yat-sen University, Guangzhou, Guangdong, P. R. China; Department of Radiology, The Sixth Affiliated Hospital, Sun Yat-sen University, Guangzhou, Guangdong, P. R. China; Guangdong Provincial Key Laboratory of Colorectal and Pelvic Floor Diseases, Guangdong Research Institute of Gastroenterology, The Sixth Affiliated Hospital, Sun Yat-sen University, Guangzhou, Guangdong, P. R. China; Biomedical Innovation Center, The Sixth Affiliated Hospital, Sun Yat-sen University, Guangzhou, Guangdong, P. R. China; Department of Radiology, The First Affiliated Hospital of Zhengzhou University, Zhengzhou, Henan, P. R. China; School of Public Health (Shenzhen), Shenzhen Campus of Sun Yat-sen University, Shenzhen, Guangdong, P. R. China; Department of Radiology, The Sixth Affiliated Hospital, Sun Yat-sen University, Guangzhou, Guangdong, P. R. China; Guangdong Provincial Key Laboratory of Colorectal and Pelvic Floor Diseases, Guangdong Research Institute of Gastroenterology, The Sixth Affiliated Hospital, Sun Yat-sen University, Guangzhou, Guangdong, P. R. China; Biomedical Innovation Center, The Sixth Affiliated Hospital, Sun Yat-sen University, Guangzhou, Guangdong, P. R. China; Department of Radiology, The Sixth Affiliated Hospital, Sun Yat-sen University, Guangzhou, Guangdong, P. R. China; Guangdong Provincial Key Laboratory of Colorectal and Pelvic Floor Diseases, Guangdong Research Institute of Gastroenterology, The Sixth Affiliated Hospital, Sun Yat-sen University, Guangzhou, Guangdong, P. R. China; Biomedical Innovation Center, The Sixth Affiliated Hospital, Sun Yat-sen University, Guangzhou, Guangdong, P. R. China; Department of Radiology, The Sixth Affiliated Hospital, Sun Yat-sen University, Guangzhou, Guangdong, P. R. China; Guangdong Provincial Key Laboratory of Colorectal and Pelvic Floor Diseases, Guangdong Research Institute of Gastroenterology, The Sixth Affiliated Hospital, Sun Yat-sen University, Guangzhou, Guangdong, P. R. China; Biomedical Innovation Center, The Sixth Affiliated Hospital, Sun Yat-sen University, Guangzhou, Guangdong, P. R. China

**Keywords:** locally advanced rectal cancer, texture analysis, apparent diffusion coefficient

## Abstract

**Background:**

Neoadjuvant chemotherapy (NCT) alone can achieve comparable treatment outcomes to chemoradiotherapy in locally advanced rectal cancer (LARC) patients. This study aimed to investigate the value of texture analysis (TA) in apparent diffusion coefficient (ADC) maps for identifying non-responders to NCT.

**Methods:**

This retrospective study included patients with LARC after NCT, and they were categorized into nonresponse group (pTRG 3) and response group (pTRG 0–2) based on pathological tumor regression grade (pTRG). Predictive texture features were extracted from pre- and post-treatment ADC maps to construct a TA model using RandomForest. The ADC model was developed by manually measuring pre- and post-treatment ADC values and calculating their changes. Simultaneously, subjective evaluations based on magnetic resonance imaging assessment of TRG were performed by two experienced radiologists. Model performance was compared using the area under the curve (AUC) and DeLong test.

**Results:**

A total of 299 patients from two centers were divided into three cohorts: the primary cohort (center A; *n *=* *194, with 36 non-responders and 158 responders), the internal validation cohort (center A; *n *=* *49, with 9 non-responders) and external validation cohort (center B; *n *=* *56, with 33 non-responders). The TA model was constructed by post_mean, mean_change, post_skewness, post_entropy, and entropy_change, which outperformed both the ADC model and subjective evaluations with an impressive AUC of 0.997 (95% confidence interval [CI], 0.975–1.000) in the primary cohort. Robust performances were observed in internal and external validation cohorts, with AUCs of 0.919 (95% CI, 0.805–0.978) and 0.938 (95% CI, 0.840–0.985), respectively.

**Conclusions:**

The TA model has the potential to serve as an imaging biomarker for identifying nonresponse to NCT in LARC patients, providing a valuable reference for these patients considering additional radiation therapy.

## Introduction

Neoadjuvant therapy is the recommended standard treatment for patients with locally advanced rectal cancer (LARC) prior to total mesorectal excision (TME) [1]. The primary objective of neoadjuvant therapy is to achieve preoperative tumor regression; however, there remains a lack of consensus regarding the selection of a neoadjuvant treatment regimen. The FOWARC study has demonstrated that neoadjuvant-modified FOFLOX6, with or without radiation, can yield comparable treatment outcomes in terms of tumor downstaging rate and 3-year disease-free survival rate [2, 3]. In addition, the PROSPECT trial also confirmed that preoperative neoadjuvant chemotherapy (NCT) was noninferior to preoperative chemoradiotherapy in terms of disease-free survival [4]. It indicates that NCT alone can achieve similar expected outcomes in terms of tumor regression while avoiding the side effects of radiation on LARC patients. Nevertheless, it is important to acknowledge that not all patients respond favorably to NCT, emphasizing the need for timely identification of nonresponsive patients for considering additional radiation therapy. Therefore, there is an urgent need to develop a predictive method for identifying non-responders to NCT prior to surgery.

Magnetic resonance imaging (MRI) is commonly utilized to assess the status of rectal cancer, which provides a comprehensive visualization of the rectum and adjacent organs, facilitating precise primary tumor staging [5]. However, MRI has inherent limitations in differentiating residual tumor from surrounding fibrosis, thereby impacting restaging after neoadjuvant therapy [6, 7]. Diffusion-weighted imaging (DWI) is a specialized functional MRI technique that detects the movement of water molecules within living tissues. The apparent diffusion coefficient (ADC), a quantitative parameter derived from DWI, provides valuable insights into the extent of water diffusion within tissues, reflecting information related to tissue cellularity [8]. The motion caused by diffusion is primarily influenced by tissue and cell properties, cell membrane integrity, and fluid viscosity [9]. Previous studies have demonstrated that ADC values and their changes can be employed to evaluate the response to neoadjuvant therapy in LARC patients [10, 11]. However, the studies have primarily focused on neoadjuvant chemoradiotherapy, with the potential influence of radiation on the ADC values and their changes.

Recently, radiomics has emerged as a promising tool for predicting prognosis and guiding therapeutic decisions by enabling high-throughput extraction of numerous imaging features from radiological images [12]. Texture analysis (TA), a burgeoning field within radiomics, facilitates objective assessments of heterogeneous target lesions and their microenvironment, which plays a crucial role in disease progression and treatment resistance [13]. As a quantitative imaging biomarker, TA enables the assessment of tumor heterogeneity by quantifying the distribution of gray-scale intensity on a pixel-by-pixel basis [14, 15]. Previous studies have demonstrated that texture features derived from T2-weighted imaging (T2WI) images can be utilized to predict the efficacy of neoadjuvant therapy in LARC patients [14–16]. In addition, Enkhbaatar *et al*. [17] have identified skewness calculated from ADC maps as a significant factor associated with a favorable response to neoadjuvant therapy in patients with LARC. However, there is currently no robust prediction model based on multiple texture features and no texture features derived from ADC maps for identifying nonresponse to NCT in LARC patients.

The objective of this study was to utilize texture features extracted from ADC maps for precise identification of nonresponse to NCT in LARC patients, thereby providing a valuable reference for non-responders considering additional radiation therapy.

## Materials and methods

This retrospective study was approved by the ethics committee of the Sixth Affiliated Hospital of Sun Yat-sen University (Guangzhou, China; center A) and the First Affiliated Hospital of Zhengzhou University (Zhengzhou, China; center B). Patient informed consent was waived due to the retrospective nature of this study.

### Eligibility criteria

**Figure 1.**
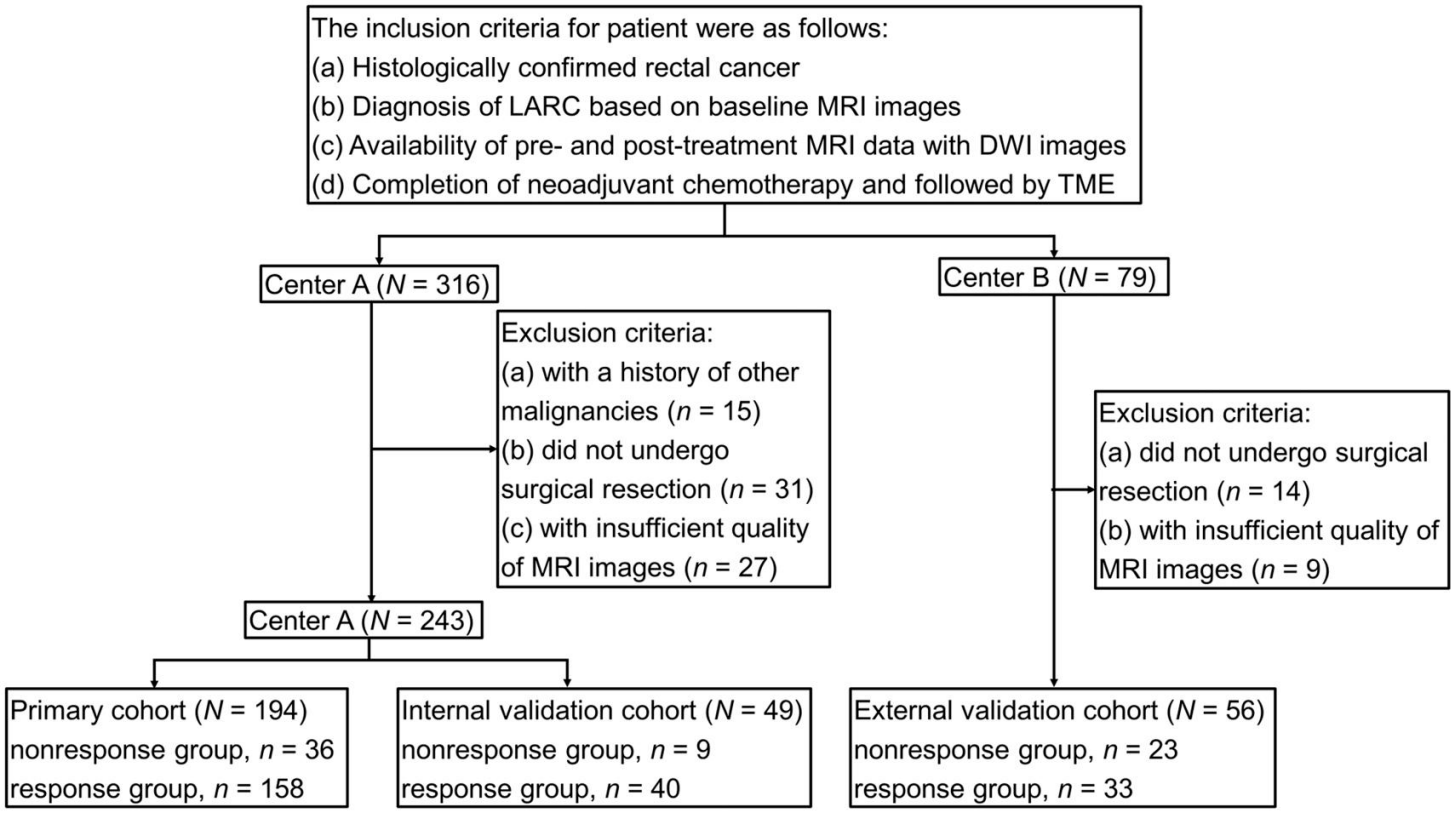
Flowchart of patient inclusion and exclusion. LARC = locally advanced rectal cancer, MRI = magnetic resonance imaging, DWI = diffusion-weighted imaging, TME = total mesorectal excision.

This study included all patients diagnosed with LARC at center A between June 2013 and January 2020, as well as those diagnosed at center B between January 2018 and August 2020. The following inclusion criteria were applied: (a) histologically confirmed rectal cancer, (b) diagnosis of LARC (T3–4N_any_ or T1–2N1–2) based on baseline MRI images, (c) availability of pre- and post-treatment MRI data with DWI images, and (d) completion of NCT (modified FOLFOX6 without radiation) according to the FOWARC clinical trial, followed by TME. Patients with a history of other malignancies, those who did not undergo surgical resection, those with insufficient quality of MRI images for evaluation, or those with histologically confirmed rectal mucinous adenocarcinoma or signet ring cell carcinoma were excluded from the study. The flowchart of patient inclusion and exclusion is shown in Figure 1.

### Primary and validation cohorts

**Figure 2.**
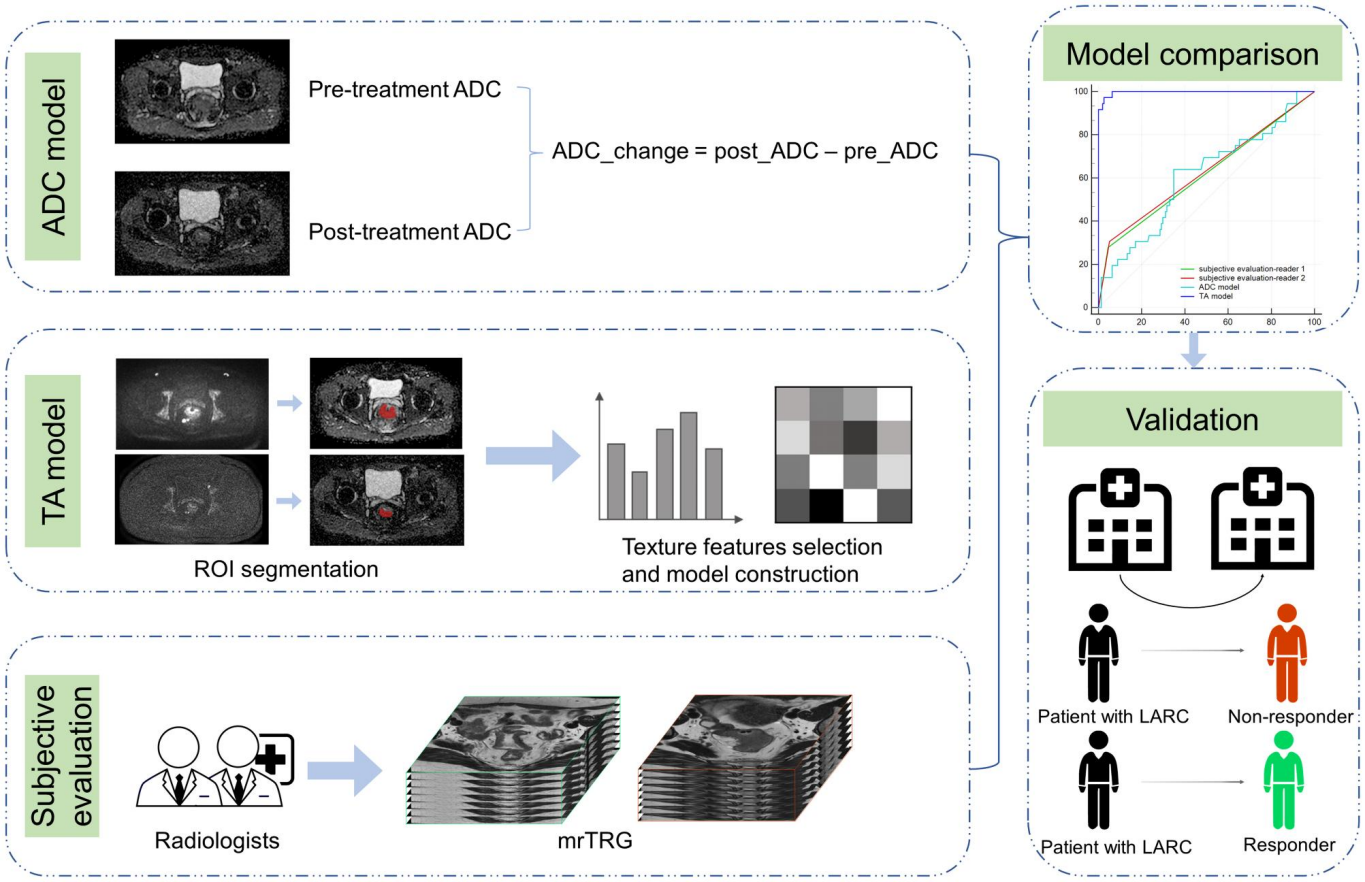
The overall experimental design in the study. ADC = apparent diffusion coefficient, TA = texture analysis, ROI = region of interest, TRG = tumor regression grade, LARC = locally advanced rectal cancer.

The patients in this study were divided into three cohorts. Patients from center A were randomly assigned to the primary cohort (*n *=* *194 patients) and the internal validation cohort (*n *=* *49 patients). The primary cohort was used for extracting texture features and constructing the TA model. The external validation cohort (*n *=* *56 patients) from center B, equipped with different equipment, was utilized for external validation. The overall experimental design is shown in Figure 2.

### MRI protocol

MRI images of patients from center A were performed with a 1.5-T MR unit (Optimal 360, GE Healthcare, Waukesha, Wis), and MRI images of patients from center B were obtained with a 3.0-T MR unit (MR 750w, GE Healthcare, Waukesha, Wis). The rectal MRI protocol in center A included oblique axial, coronal, and sagittal T2-weighted imaging, oblique axial T1-weighted imaging, diffusion-weighted imaging, and gadolinium-enhanced T1-weighted imaging, as summarized in Supplementary Table S1. The ADC maps were generated by automatically fusing DWI images at b-values of 0 and 1,200 s/mm^2^ using a GE AW 4.5 post-processing workstation.

### Subjective evaluations

Two radiologists (reader 1 and reader 2, with 10–20 years of experience in gastrointestinal diagnosis) were blinded to pathological outcomes and clinical data, and independently evaluated all pre- and post-NCT MRI images in the primary cohort. MRI tumor regression grade (mrTRG) was assessed according to the method established by the MERCURY study group [18]. Patients were categorized into two groups based on mrTRG: nonresponse group (characterized by the absence of any signs of regression or a radiological appearance similar to pre-NCT) and response group (presented with signs of tumor regression).

### ADC values acquisition and ADC model construction

The pre- and post-treatment T2WI and DWI images were carefully examined to identify the primary tumor, characterized by medium to a high signal intensity corresponding to the location of the tumor mass. In cases where a significant reduction in tumor volume was observed after treatment, the signal intensity on DWI images might also decrease, manifesting as low signal intensity. In such instances, the T2WI sequences were used as a reference to confirm the lesion of the primary tumor [19]. Another two radiologists (reader 3 and reader 4, with 5 and 10 years of experience in gastrointestinal diagnosis, respectively) manually positioned the region of interest (ROI) on both pre- and post-treatment DWI images (with a high b value) using a GE AW 4.5 post-processing workstation. The ROIs were then transferred to the corresponding ADC maps. The delineation was performed on a single trans-axial slice containing the largest tumor area, avoiding the intestinal lumen and necrotic area. Each radiologist independently reviewed the pre- and post-treatment DWI images for ADC measurements. The averaged ADC values from two radiologists were labeled as the ADC for subsequent data analysis. The pre-treatment and post-treatment ADC values were designated as pre_ADC and post_ADC, respectively. ADC_change was calculated by subtracting the pre_ADC from the post_ADC. The ADCs with statistical differences were incorporated into the logistic regression analysis utilizing the Likelihood Ratios (LR) forward method to establish an ADC model.

### Texture features implementations and TA model construction

An experienced radiologist (reader 1) performed image analysis by integrating T2WI and DWI images to precisely identify the tumor area in the ADC maps. The itk-SNAP platform (www.itksnap.org) was utilized for meticulous manual delineation of the tumor outline within the largest cross-sectional area of ADC maps, avoiding the intestinal lumen and necrotic area, which was designated as the tumor ROI. Subsequently, another experienced radiologist (reader 2) conducted a comprehensive review of the initial ROI delineation to ensure accuracy and consistency. In instances where any errors or labeling discrepancies in tumor determination were identified, prompt communication and discussion took place between reader 1 and reader 2 to establish a consensus. The adjusted ROI was then employed for subsequent analyses.

The images from the two centers were subjected to normalization techniques for processing. Subsequently, texture features were meticulously extracted from the above designated areas using Matlab2016b software (MathWorks, Natick, MA) by a researcher who was blinded to the pathological outcomes. The spatial distribution of the ADC value was characterized by texture features. Various texture feature parameters were calculated, including mean, standard deviation (SD), variance, entropy, skewness, and kurtosis coefficients. Those parameters were employed to provide insights into the disorder, symmetry, and boundary behavior of the distribution of signal intensity within the MRI images [14, 20]. Details of the texture features are summarized in Supplementary Table S2. After two months, both reader 1 and reader 2 independently re-delineated ROIs for 100 randomly selected patients. The interobserver correlation coefficient (ICC) was used to assess measurement reproducibility. Texture features with an ICC > 0.75 were considered to exhibit good agreement in reproducibility.

In terms of feature selection, a random forest classifier was used to identify the predictive features, with the Gini index as an evaluation criterion. Subsequently, the TA model based on RandomForest was generated using sklearn (version 1.2.1, https://scikit-learn.org/).

### Pathological assessment of response

The surgical specimens underwent pathological analysis according to the methods outlined in the 7th edition of the American Joint Committee on Cancer (AJCC) TNM staging system and the protocols described by Ryan *et al*. [21, 22]. Subsequently, all patients were categorized based on their treatment response using the pathological tumor regression grade (TRG) system [23]. This system classifies patients into four TRG groups as follows: TRG 0, no residual tumor cells; TRG 1, single tumor cell or small group of tumor cells; TRG 2, residual cancer with desmoplastic response and TRG 3, no definitive response. Patients were then divided into a nonresponse group (pTRG 3) and a response group (pTRG 0–2).

### Statistical analysis

The χ^2^ test or Fisher's exact test was used to analyze categorical variables between nonresponse and response groups, while the student’s *t*-test or Mann-Whitney *U* test was utilized for analyzing continuous variables, including texture features and ADC values.

Receiver operating characteristic (ROC) analyses were applied to evaluate the diagnostic accuracy of subjective evaluations, ADC model, and TA model by calculating the area under the curve (AUC). Subsequently, AUC values were compared using the DeLong test. In addition, ROC analyses were conducted in both internal and external validation cohorts to ensure the reliability and consistency of the TA model.

Statistical analyses were performed using IBM SPSS Statistics (version 26.0, NY) and MedCalc Software (version 20.0, Belgium). A *P* value of < 0.05 was considered a statistically significant difference.

## Results

### Demographic and clinical characteristics

In this study, we enrolled 299 patients who underwent standard NCT, including 68 (22.7%) non-responders and 231 (77.3%) responders. The primary cohort consisted of 158 (81.4%) responders and 36 (18.6%) non-responders, whereas the internal validation cohort included 40 (81.6%) responders and 9 (18.4%) non-responders. In the external validation cohort, there were 23 (41.1%) responders and 33 (58.9%) non-responders. Notably, there were no significant differences observed in clinical variables between nonresponse group and response group in both the primary and internal validation cohorts. The comprehensive details regarding the study participants are presented in Table 1.

**Table 1.** Demographic and clinical characteristics in the primary and internal validation cohorts

Characteristic	Primary cohort (*n *=* *194)		*P*	Internal validation cohort (*n *=* *49)		*P*
	Nonresponse group (*n *=* *36)	Response group (*n *=* *158)		Nonresponse group (*n *=* *9)	Response group (*n *=* *40)	
Age, years, mean ± SD	49.42 ± 14.20	54.22 ± 11.95	0.066	54.22 ± 7.98	54.33 ± 13.62	0.111
Gender, *n* (%)			0.330			0.415
Male	28 (77.78)	110 (69.62)		8 (88.89)	27 (67.50)	
Female	8 (22.22)	48 (30.38)		1 (11.11)	13 (32.50)	
Pre-NCT T-staging, *n* (%)			0.413			0.264
T2	3 (8.33)	12 (7.59)		0 (0)	3 (7.50)	
T3	29 (80.56)	114 (72.15)		5 (55.56)	29 (72.50)	
T4	4 (11.11)	32 (20.25)		4 (44.44)	8 (20.00)	
Pre-NCT N-staging, *n* (%)			0.104			0.272
N0	4 (11.11)	43 (27.21)		4 (44.44)	8 (20.00)	
N1	18 (50.00)	58 (36.71)		2 (22.22)	17 (42.50)	
N2	14 (38.89)	57 (36.08)		3 (33.33)	15 (37.50)	

NCT = neoadjuvant chemotherapy, SD = standard deviation.

### Texture features between nonresponse group and response group in the primary cohort

The TA features between non-responders and responders are summarized in Table 2. Significant statistical differences were observed in post_mean, mean_change, post_variance, post_entropy, and post_SD between nonresponse group and response group in the primary cohort (*P *<* *0.05). The post-treatment skewness and kurtosis were smaller than those of pre-treatment, while the other parameters showed an increase after NCT.

**Table 2.** The ADC values and texture features

Variable	Nonresponse group (*n *=* *36)	Response group (*n *=* *158)	*P*
ADC value (10^−3^ mm^2^/s)			
pre_ADC	0.961 ± 0.229	1.018 ± 0.236	0.192^a^
post_ADC	0.947 ± 0.283	1.043 ± 0.256	0.048^a^
ADC_change	−0.014 ± 0.220	0.025 ± 0.190	0.018^b^
Texture features			
pre_mean	0.962 ± 0.229	1.030 ± 0.221	0.101^a^
post_mean	0.906 ± 0.269	1.060 ± 0.240	<0.001^a^
mean_change	−0.056 ± 0.125	0.030 ± 0.177	<0.001^b^
pre_variance	150.680 ± 52.904	162.902 ± 58.214	0.282^b^
post_variance	169.219 ± 55.712	201.836 ± 73.256	0.025^b^
variance_change	18.540 ± 57.628	38.934 ± 80.590	0.120^b^
pre_skewness	0.946 ± 0.715	0.811 ± 0.687	0.322^b^
post_skewness	0.405 ± 0.873	0.293 ± 0.690	0.174^b^
skewness_change	−0.541 ± 1.238	−0.518 ± 0.913	0.633^b^
pre_kurtosis	1.685 ± 2.922	1.104 ± 2.117	0.339^b^
post_kurtosis	1.133 ± 3.212	0.325 ± 1.233	0.068^b^
kurtosis_change	−0.552 ± 3.741	−0.779 ± 2.229	0.608^b^
pre_entropy	−5.108 ± 0.302	−5.071 ± 0.309	0.599^b^
post_entropy	−5.054 ± 0.273	−4.828 ± 0.463	0.002^b^
entropy_change	0.054 ± 0.392	0.244 ± 0.529	0.097^b^
pre_SD	12.081 ± 2.208	12.560 ± 2.279	0.254^a^
post_SD	12.813 ± 2.277	14.000 ± 2.425	0.025^b^
SD_change	0.733 ± 2.494	1.440 ± 2.821	0.167^a^

Data are means ± standard deviation.

a *P*-values were determined by using student’s *t*-test;.

b Others were using the Mann-Whitney *U* test.

ADC = apparent diffusion coefficient, SD = standard deviation.

### Comparison between subjective evaluations, ADC model, and TA model

In the primary cohort, there was poor agreement observed between the two radiologists in their assessments of nonresponse to NCT, with a kappa value of 0.265 (95% confidence interval [CI], 0.054–0.477). Reader 1 demonstrated a sensitivity of 27.8% and specificity of 95.6%, while reader 2 showed a sensitivity of 30.6% and specificity of 94.9%. The AUCs for subjective evaluations by both readers were 0.617 (95% CI, 0.544–0.685) and 0.627 (95% CI, 0.555–0.696), respectively, with no significant difference observed between them (*P *=* *0.836) (Table 3).

**Table 3.** ROC analyses of different evaluation methods in the primary cohort

Evaluation method	Sensitivity (%)	Specificity (%)	AUC	DeLong test^a^
TA model	97.2	97.5	0.997 (0.975–1.000)	–
Subjective evaluation				
reader 1	27.8	95.6	0.617 (0.544–0.685)	<0.001
reader 2	30.6	94.9	0.627 (0.555–0.696)	<0.001
ADC model	63.9	65.2	0.607 (0.534–0.676)	<0.001

a Comparison of subjective evaluation or ADC model with TA model using DeLong test. There was no statistically significant difference between the ADC model and subjective evaluations conducted by reader 1 and reader 2 (*P *=* *0.864 and 0.723, respectively).

ROC = receiver operating characteristic, AUC = the area under the curve, TA = texture analysis, ADC = apparent diffusion coefficient.

The pre_ADC did not exhibit a statistically significant difference between nonresponse and response groups (*P *=* *0.192). Specifically, a decrease in ADC values was observed in nonresponse group after NCT. The post_ADC and ADC_change, which showed statistically significant differences between nonresponse and response groups (*P *=* *0.048 and 0.018, respectively) (Table 2), were subsequently incorporated into the logistic regression model; however, only post_ADC remained significant in the ADC model. The AUC value of the ADC model for predicting non-responders was 0.607 (95% CI, 0.534–0.676) (Table 3) in the primary cohort. Nevertheless, there were no statistically significant differences between the ADC model and subjective evaluations conducted by reader 1 and reader 2 (*P *=* *0.864 and 0.723, respectively).

**Figure 3.**
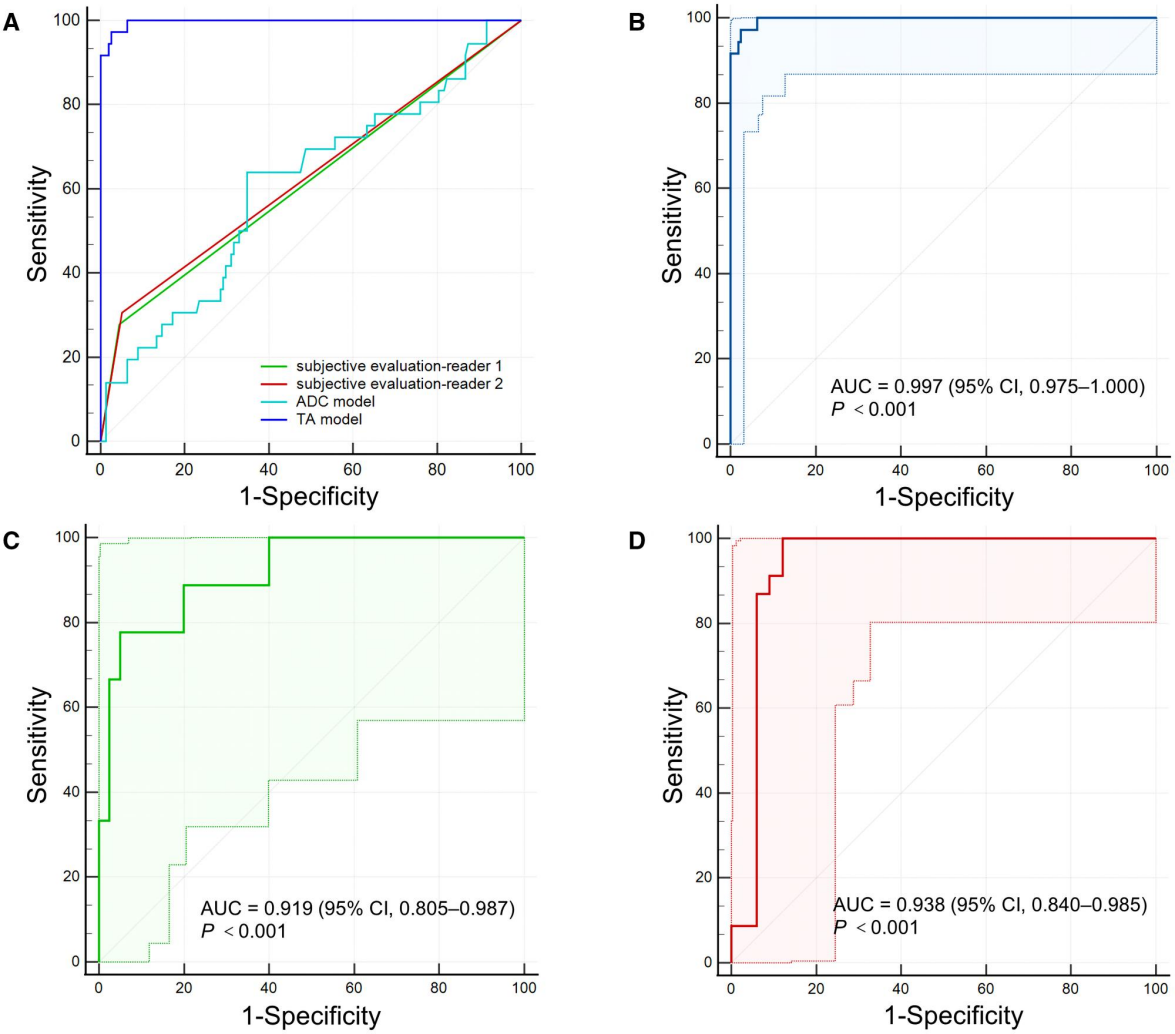
The ROC analysis results. (A) Different models; (B) TA model in the primary cohort; (C) internal validation cohort; (D) external validation cohort. ROC = receiver operating characteristic, TA = texture analysis.

The TA model was constructed by post_mean, mean_change, post_skewness, post_entropy, and entropy_change, which outperformed both the ADC model and subjective evaluations with an impressive AUC of 0.997 (95% CI, 0.975–1.000) (*P *<* *0.001) (Table 3). Furthermore, the robust performance of the TA model was demonstrated in both internal and external validation cohorts, with AUCs of 0.919 (95% CI, 0.805–0.978) and 0.938 (95% CI, 0.840–0.985), respectively (Figure 3).

## Discussion

Our research has successfully developed a predictive model based on texture features extracted from ADC maps, enabling accurate identification of LARC patients with nonresponse to NCT. Remarkably, our model exhibited robust and superior performance with AUCs of 0.997, 0.919, and 0.938 in the primary, internal validation, and external validation cohorts, respectively. This model provides valuable radiological evidence that significantly contributes to the consideration of additional radiation therapy in LARC patients with nonresponse to NCT.

Preoperative chemoradiotherapy for patients diagnosed with LARC is administered before major surgery, serving the dual purpose of optimizing patient compliance and facilitating tumor down-staging. This approach not only holds the potential to improve the surgical cure rate but also allows for sphincter preservation, particularly in cases involving low-lying tumors [24]. It has been well-documented that preoperative administration of chemoradiotherapy significantly prolongs disease-free survival compared to postoperative treatment, with a trend suggesting improved overall survival [25]. However, individual variations in response to preoperative chemoradiotherapy can result in both unnecessary patient toxicity and treatment delays when ineffectiveness occurs [26]. Deng *et al*. [2, 27] pointed out that preoperative mFOLFOX6 alone can achieve comparable downstaging rates and disease-free survival as fluorouracil-radiation, while exhibiting lower toxicity and fewer postoperative complications. Therefore, our research focused on identifying patients who were nonresponse to NCT, which carried significant clinical implications for tailoring more effective treatment strategies including the consideration of additional radiation therapy.

Several pathological TRG systems have been developed based on the relative proportion of fibrosis observed in resection specimens [28], which has been established as a surrogate marker for individual-level disease-free survival [29]. The application of similar principles in MRI, as demonstrated by the MERCURY study, highlights the utility of mrTRG in preoperative evaluating tumor regression [18]. While mrTRG is a valuable tool for evaluating tumor response to therapy, its limitations stem from its reliance on visual and qualitative assessment of signal intensity characteristics in T2WI and DWI images, potentially introducing subjectivity and interobserver variability. In our study, we observed a significant lack of agreement in the subjective evaluations, with the sensitivity of two experienced radiologists being less than 40%. This finding has the potential to impede the effective utilization of mrTRG in our research. The primary challenge arises from the similarity in signal intensity characteristics on T2WI and DWI images before and after NCT for tumors classified as mrTRG 2 and mrTRG 3.

DWI is a noninvasive imaging technique that relies on the Brownian motion of water molecules and holds significance in the early diagnosis of rectal cancer [30]. The ADC, derived from DWI images, serves as a quantitative parameter for evaluating an array of molecular properties, including cell density, vascularity, viscosity of extracellular fluid, and cell membrane integrity [9, 31]. Moreover, ADC values exhibit an inverse relationship with cell density, where high cell densities manifest as low ADC values owing to restricted water movement within high tissue confinement [32, 33]. Accordingly, it has been consistently observed that effective treatment in LARC leads to an increase in ADC values. Genovesi *et al*. [34] pointed out that the increase in ADC value among LARC patients achieving complete response was greater compared to those in the non-complete response group after neoadjuvant chemoradiotherapy. In addition, the changes in ADC values before and after neoadjuvant therapy were found to be correlated with TRG [17]. However, despite the predominant trend of increased ADC values following therapy reported by most studies, our findings revealed a decrease in ADC values among patients in nonresponse group after treatment. This suggests that non-responders may exhibit elevated tumor activity and heterogeneity, with some individuals experiencing tumor progression after NCT.

An increasing number of studies have adopted TA to evaluate tumor heterogeneity and assess the predictive value of texture features in preoperative chemoradiotherapy response among LARC patients [13, 35]. Gourtsoyianni *et al*. [15] have demonstrated the repeatability and sufficiently robust performance of texture features for clinical application in rectal cancer. Caruso et al. [36] found lower entropy in pretreatment T2WI images was observed among patients with complete response. De Cecco *et al*. [16] revealed that pretreatment kurtosis is the most effective parameter for predicting tumor response, showing sensitivity and specificity for pathological complete response detection of 100% and 77.8%, respectively. Similarly, other scholars have also proposed that posttreatment entropy can be utilized to identify individuals with a complete response in LARC after neoadjuvant treatment [14]. In our study, we employed RandomForest to construct the TA model, which primarily incorporated texture features from the post-treatment images. We hypothesized this phenomenon may be attributed to the increased heterogeneity within the primary tumor site after NCT, resulting from a complex interplay of residual tumor, treatment-induced fibrosis, edema, and necrosis. In clinical practice, these various tissue components within the primary tumor site exhibit distinct radiographical signal characteristics in MRI images.

There are several **limitations** in this study. First, the potential for selection bias due to the retrospective design of our study should be taken into consideration. Second, the tumor segmentation was conducted on the largest cross-sectional area of the tumor rather than the entire tumor, which may provide a more representative assessment of tumor heterogeneity. Third, it was challenging to place the ROIs properly, particularly in cases where subtle residual tumors were identified on DWI images after neoadjuvant therapy. We made efforts to combine pretreatment and posttreatment images and incorporated multiple sequences to ensure precise identification of residual tumors. Finally, the immune microenvironment or protein expression is closely related to tumor heterogeneity, impacting treatment efficacy and prognosis [37]. Investigating the correlation between texture features and protein expression or the immune microenvironment is an avenue for future research.

In conclusion, texture features derived from ADC maps could serve as valuable imaging biomarkers for identifying LARC patients with nonresponse to NCT. Our findings suggest that the TA model based on ADC maps could effectively identify patients who have nonresponse to NCT, thereby facilitating these patients in considering additional radiation therapy before surgery.

## Supplementary Material

goae035_Supplementary_Data
