# Supplementary material for: Texture analysis of apparent diffusion coefficient maps: can it identify nonresponse to neoadjuvant chemotherapy for additional radiation therapy in rectal cancer patients?
Source: Gastroenterol Rep (Oxf). 2024 Apr 22;12:goae035. doi: 10.1093/gastro/goae035 (PMC11035003; doi:10.1093/gastro/goae035)
Supplement: goae035_Supplementary_Data [file goae035_supplementary_data.zip › 2023-505 Supplementary Table_S2.docx]

**Supplementary Table S2. The definition and implication of texture features**

| **Feature** | **Definition** | **Implication** |
| --- | --- | --- |
| mean | average intensities of pixels within ROI | Higher value means a whiter image. |
| variance | mean of the squared distances of each intensity value from the Mean value. | A low Variance means that distribution of intensities is close to the mean. A high Variance means that intensities are spread out among a large range of values. |
| SD | measurement of dispersion from the average | A low SD means that distribution of intensities is close to the mean. A high SD means that intensities are spread out among a large range of values. |
| skewness | measurement of the asymmetry of the histogram | Positive skewness: indicates that the right tail of the histogram is longer than the left side. Negative skewness: indicates that the left tail of the histogram is longer than the right side. A zero value means the values are evenly distributed on either side of the mean. (normal distribution) |
| kurtosis | measurement of peakedness of the histogram | Positive kurtosis: distribution is more peaked than the normal distribution. Negative kurtosis: distribution is flatter than the normal distribution. Value of 3 is normal distribution. |
| entropy | measurement disorder in the distribution of intensities | Higher values mean more chaos. Lower values mean more homogeneity. |

Note. ROI = region of interest, SD = standard deviation.
