# Supplementary material for: Texture analysis of apparent diffusion coefficient maps: can it identify nonresponse to neoadjuvant chemotherapy for additional radiation therapy in rectal cancer patients?
Source: Gastroenterol Rep (Oxf). 2024 Apr 22;12:goae035. doi: 10.1093/gastro/goae035 (PMC11035003; doi:10.1093/gastro/goae035)
Supplement: goae035_Supplementary_Data [file goae035_supplementary_data.zip › 2023-505 Supplementary Table_S1.docx]

Supplementary **Table S1. The technical MRI parameters of the scanning sequences**

| Plane | TR/TE (ms) | NEX | FOV (cm) | Matrix | Section Thickness/Gap (mm) |
| --- | --- | --- | --- | --- | --- |
| Oblique Axial T2WI | 4294/108 | 4 | 24 × 24 | 288 × 256 | 3/0.5 |
| Oblique Coronal T2WI | 2358/108 | 4 | 24 × 24 | 288 × 256 | 3/0.5 |
| Oblique Sagittal T2WI | 2591/125 | 4 | 28 × 28 | 288 × 224 | 3/0.5 |
| Oblique Axial DWI  (b = 0, 1200 s/mm^2^) | 4550/92.6 | 4 | 40 × 40 | 192 × 192 | 3/0.5 |
| Oblique Axial T1WI | 419/13.5 | 2 | 24 × 24 | 320 × 224 | 3/0.5 |

MRI = magnetic resonance imaging, TR = repetition time, TE = echo time, NEX = number of excitation, FOV = field of view, T2WI = T2-weighted imaging. DWI = diffusion-weighted imaging, T1WI = T1-weighted imaging.
